# Anti‐Inflammatory/Antioxidant Features of Adipose Tissue Mesenchymal Stem Cells Conditioned Media for Doped TiO_2_ Nanoparticles in Induced Inflammation

**DOI:** 10.1002/open.202500261

**Published:** 2025-06-09

**Authors:** Ahmed A. Abd‐Rabou, Ahmed M. Youssef, Mohamed I. El‐Khonezy, Soheir E. Kotob, Mohamed S. Kishta

**Affiliations:** ^1^ Hormones Department Medical Research and Clinical Studies Institute National Research Centre Dokki Cairo 12622 Egypt; ^2^ Stem Cell Lab. Center of Excellence for Advanced Sciences National Research Centre Dokki Cairo 12622 Egypt; ^3^ Inorganic Chemistry Department Inorganic Chemical Industries and Mineral Resources Research Institute National Research Centre Dokki Cairo 12622 Egypt; ^4^ Molecular Biology Department Biotechnology Research Institute National Research Centre Dokki Cairo 12622 Egypt

**Keywords:** adipose tissue mesenchymal stem cells, antioxidants, doped TiO_2_, inflammation, lipopolysaccharide

## Abstract

The fundamental purpose of this work is to determine the anti‐inflammatory/antioxidant activity of stem cell conditioned medium enhanced by mono‐ or dual‐doped TiO_2_ nanoparticles. The transmission electron microscope, X‐ray diffraction, and energy‐dispersive X‐ray analyses are used to characterize the nanoparticles. Isolation/characterization of adipose tissue mesenchymal stem cells (AD‐MSCs) is done. In vitro assays reveal that protein denaturation and proteinase induction are significantly increased with lipopolysaccharide (LPS) comparable to control, while treatments significantly decrease the induction compared to LPS. In vitro assays reveal decreasing in hydroxyl radical scavenging and DPPH radical scavenging activities with LPS, while treatments significantly increase the activity compared to LPS. Induction with LPS decreases in vitro catalase (CAT), superoxide dismutase (SOD), and glutathione peroxidase (GPx) expression levels and enzyme activities are significantly compared to controls, while treatments significantly increase the CAT expression compared to LPS. Induction with LPS increases the in vitro interleukin 4 (IL4), IL6, IL10, and tumor necrosis factor α expression levels, and their activities significantly decline with treatment compared to controls, while treatments significantly decline expression compared to LPS. The anti‐inflammatory and antioxidant properties of AD‐MSC‐conditioned medium enhanced with mono‐ or dual‐doped TiO_2_ nanoparticles are identified in this investigation. To sum up, the work has shown that mono‐ and dual‐doped TiO_2_ can inhibit the inflammation caused by LPS in vitro.

## Graphical abstract

The PI3K/AKT pathway is triggered when lipopolysaccharide (LPS) binds to TLR4 on the cell membrane. This causes the NF‐κB complex to get phosphorylated and IKB to degrade, allowing NF‐κB to go into the nucleus. Pro‐inflammatory genes including interleukin‐4 (IL‐4), IL‐6, and tumor necrosis factor α are triggered by this. NF‐κB activation is further enhanced by LPS's simultaneous induction of oxidative stress, which produces reactive oxygen species (ROS) and byproducts like MDA. By breaking down the Keap1‐Nrf2 complex, ROS releases Nrf2, which moves to the nucleus and attaches to the antioxidant response element to promote the production of antioxidant enzymes including superoxide dismutase (SOD), catalase (CAT), and glutathione peroxidase (GPx). This activates the Nrf2 pathway to combat oxidative. The addition of TiO_2_ nanoparticles (mono‐ and dual‐doped) and adipose tissue mesenchymal stem cell (AD‐MSC)‐conditioned media modulates these pathways by reducing oxidative and inflammation, with AD‐MSC bioactive factors and TiO_2_ nanoparticles (NPs) synergistically enhancing the anti‐inflammatory and antioxidant responses to restore cellular balance.

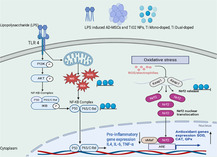

## 1 Introduction

Stem cells have received a lot of attention in recent decades because of their special qualities, such as their capacity to self‐replicate and specialize into a variety of cell types,^[^ ^1^, ^2^ ^]^ which makes them a prime prospect for uses in regenerative medicine including their ability to self‐reproduce.^[^ ^3^ ^]^ Adipose‐derived mesenchymal stromal cells (AD‐MSCs) generated from adipose tissue possess both immunomodulatory and regenerative properties, making them a promising therapeutic alternative for symptomatic relief from inflammation and oxidative.^[^ ^4^ ^]^ AD‐MSCs have demonstrated paracrine activities that prevent monocyte and myeloid dendritic cell growth as well as limit the production of inflammatory T‐cells.^[^ ^5^, ^6^ ^]^ In particular, studies conducted in vitro suggest that AD‐MSCs may directly lower chondrocyte inflammation and the production of mediators such tumor necrosis factor α (TNF‐α).^[^ ^7^ ^]^ Through alterations in cytokine expression and direct cell‐to‐cell communication, MSCs can also influence the production of extracellular matrix and chondrocyte differentiation.^[^ ^8^ ^]^

Preclinical model research together indicates that intra‐articular AD‐MSCs injection may improve functional symptoms. Stem cell conditioned media (CM), which is defined as the cell‐free media containing the secreted factors or secretome of stem cells, has been shown to exhibit regenerative capacity.^[^ ^9^ ^]^ Their involvement might be linked to their anti‐inflammatory capabilities including suppression of T cell proliferation and activation of anti‐inflammatory cytokines like interleukins. They also exhibit antioxidant potential as indicated by their ability to boost the activities of antioxidant enzymes such as glutathione peroxidase (GPx), catalase (CAT), and superoxide dismutase (SOD).^[^ ^10^ ^]^

To enhance the anti‐inflammatory and antioxidant ability of the CM of the AD‐MSCs, in the current work, we fed it with titanium dioxide nanoparticles, mono‐doped titanium dioxide nanoparticles with copper, and dual‐doped titanium dioxide nanoparticles with copper and zinc. An lipopolysaccharide (LPS)‐induced cell model was developed to study these titanium dioxide‐based nanoparticles. The LPS, a component of the bacterial outer membrane, was used to induce inflammation. Up to seven acyl chains that makeup “lipid A,” which is attached to an oligosaccharide central and an extremely inconstant polysaccharide chain called O‐antigen, anchor LPS in the bacterial membrane. The greatest evolving preserved component of LPS, lipid A, is what gives it its pro‐inflammatory properties. LPS has the greatest ability to cause inflammation because it has a bis‐phosphorylated lipid A that is made up of six saturated acyl chains. Although excessive host reactions to LPS can result in systemic inflammatory diseases such as sepsis and deadly septic shock, LPS plays a critical role in reducing bacterial infections.^[^ ^11^, ^12^, ^13^ ^]^

The capacity of titanium dioxide to block pro‐inflammatory mediators like IL6 and decrease inflammation in LPS‐induced RAW264.7 cells has demonstrated its anti‐inflammatory qualities.^[^ ^14^ ^]^ An intriguing option for antioxidant and anti‐inflammatory uses is copper. It has shown tremendous promise when reduced to nanotubes, including the capacity to maintain the cell membrane, prevent albumin denaturation, and minimize the synthesis of inflammatory mediators at the molecular level.^[^ ^15^ ^]^ Zinc, an essential element for metabolism, has an important role in combating inflammation and oxidation, as evidenced by the amplified manufacture of reactive oxygen species (ROS) in populations that had zinc deficiency as well as decreased lipid peroxidation products in people receiving zinc supplementation. Zinc deficiency also causes inflammation produces high levels of ROS in chronic situations and encourages production of pro‐inflammatory cytokines.^[^ ^16^ ^]^

Important ROS examples are malondialdehyde (MDA) and nitric oxide (NO). MDA is a responsive aldehyde which able to connect to different molecules such as DNA, lipids, or proteins to generate new structures recognized as MDA epitopes.^[^ ^17^ ^]^ Lipid peroxidation end products have been found in inflammatory pathologies such as cardiovascular, pulmonary, hepatic, and neurological illnesses, indicating that enhanced lipid peroxidation is associated with many of these conditions.^[^ ^18^ ^]^ Moreover, NO is a dangerous signal that needs to be neutralized by both innate and adaptive immune systems.^[^ ^19^, ^20^ ^]^

Certain antioxidant enzymes, such glutathione peroxidase (GPx), catalase (CAT), and superoxide dismutase (SOD), are basically monitored because they can prevent oxidative and shield proteins and lipids from oxidation. Oxidative is reflected in a decrease in these enzymes’ output.^[^ ^21^ ^]^ Furthermore, 5‐lipoxygenase (5‐LOP) and total antioxidant capacity (TAC) must be examined in relation to inflammatory processes.^[^ ^22^, ^23^ ^]^ Thus, discovering new therapeutics that can raise the enzyme activities and TAC would be great as antioxidants. Interleukins are documented for their part in cell communication and their participation in inflammatory response. IL4 and IL6 in addition to TNF‐α are proinflammatory and contribute to inflammation, and thus a decrease in their production reflects anti‐inflammatory activity.^[^ ^24^ ^]^

This study aims to explore the capability of mono‐ and dual‐doped titanium dioxide to suppress LPS‐induced inflammatory responses through modulation of ROS mediators, interleukins, TNF‐α, SOD, CAT, GPx, and 5‐LOP.

## 2 Experimental Section

### 2.1 Study Design

The study design involved isolating and characterizing adipose tissue stem cells (AD‐MSCs) from male Sprague Dawley rats. The five groups: (1) the control group (CT), where AD‐MSCs were treated with CM; (2) the LPS group, in which AD‐MSCs were treated with LPS dissolved in complete media to induce inflammation; (3) TiO_2_ nanoparticle group (Ti), where AD‐MSCs were treated with TiO_2_ nanoparticles suspended in CM; (4) mono‐doped TiO_2_ nanoparticle group (Ti mono), involving AD‐MSCs treated with TiO_2_Cu nanoparticles suspended in CM; and (5) dual‐doped TiO_2_ nanoparticle group (Ti dual), where AD‐MSCs were treated with TiO_2_CuZn nanoparticles suspended in CM. Cytotoxicity of these treatments was assessed using the MTT assay, and oxidative markers (MDA, NO), TAC, and enzyme‐linked immunosorbent assays (ELISA) were used to measure inflammatory and antioxidant responses. Protein denaturation and proteinase inhibitory activities were evaluated to study anti‐inflammatory effects, while antioxidant activities were analyzed using hydroxyl radical and DPPH scavenging assays. Gene expression analysis of oxidative and inflammatory markers (GPx, CAT, SOD1, IL‐4, IL‐6, and TNF‐α) was performed using quantitative reverse transcriptase polymerase chain reaction (qRT‐PCR). The study compared the effects of TiO_2_ nanoparticles, mono‐doped, and dual‐doped TiO_2_ nanoparticles on AD‐MSCs under inflammatory conditions.

### 2.2 Reagents

Phosphate‐buffered saline (PBS), 0.075% collagenase II, DMEM supplemented with 10% fetal bovine serum (FBS) and 1% penicillin‐streptomycin, titanium isopropoxide (C_12_H_28_O_4_Ti), ethanol, aqueous ammonia solution, Cu(NO_3_)_2_·3H_2_O, Zn(NO_3_)_2_·6H_2_O, 0.25% trypsin, 0.01% EDTA, lipopolysaccharide (LPS), MTT reagent, dimethyl sulfoxide (DMSO), Biodiagnostic kits for MDA, NO, and TAC, and Elabscience ELISA kits for IL‐4, IL‐6, TNF‐α, and 5‐LOP, 20 mM Tris‐HCl buffer (pH 7.4), 0.8% casein, 70% perchloric acid, 1 mM 1,10‐phenanthroline, 0.1 mM hydrogen peroxide, 1 mM iron(III) chloride, 0.3 mM DPPH, methanol, L‐ascorbic acid, RNeasy Mini kit, cDNA synthesis kit, and SYBR Green PCR Master Mix are the ingredients.

### 2.3 Isolation and Characterization of Adipose Tissue Stem Cells (AD‐MSCs)

Adipose tissue was extracted from the male Sprague Dawley rats’ inguinal fat pad (the subcutaneous area) and omentum (the belly region) while they were under general anesthesia. The adipose tissue was separated and then placed in a sterile tube containing 15 mL of PBS. For 60 min at 37 °C, enzymatic digestion was conducted using 0.075% collagenase II in a sterile, balanced salt solution while being shaken.^[^ ^25^ ^]^ Following digestion, the tissue was filtered, and centrifuged, and erythrocyte lysis buffer was used to remove the erythrocytes. The cells were put in tissue culture flasks with 10% FBS added to the DMEM. The cells were then incubated in 5% humidified CO_2_ at 37 °C for 24 h. The DMEM medium with 10% FBS and 1% penicillin‐streptomycin supplement was used to culture the adherent cells. Every two to three days, cultural media changed.^[^ ^26^ ^]^ Cells were centrifuged, resuspended in a medium containing serum, and then incubated in a 25 cm^2^ culture flask. To make sure all cells have the same morphology, cultures were multiplied up to the second passage.^[^ ^27^ ^]^ An inverted microscope was used to evaluate the morphology of isolated AD‐MSCs. Additionally, flow cytometry was used to identify cell surface markers (CD90, CD73, CD34, and CD45).^[^ ^28^ ^]^

### 2.4 Mono and Dual TiO_2_ Nanoparticle Preparation and Characterization TiO_2_ Nanoparticle Preparation

To prepare a pure TiO_2_ nanopowder, 12 mL of room‐temperature titanium isopropoxide (C_12_H_28_O_4_Ti) was mixed with 25 mL of ethanol. The precipitate was formed by adding a diluted aqueous ammonia solution dropwise while continuously stirring the resulting mixture for 0.5 h, or until pH 8. The resultant precipitate was dried, washed several times with deionized water, and then calcined at 600 °C for 3 h.^[^ ^29^ ^]^

#### 2.4.1 Mono and Dual TiO_2_ Nanoparticle Preparation

By using the same technique to produce pure TiO_2_, mono‐ and dual‐doped TiO_2_ powders with Ti0.98Cu0.02O2 and Ti0.97Cu0.015Zn0.015O2 structures were prepared. In this case, ethanol‐titanium isopropoxide (C_12_H_28_O_4_Ti) mixtures were doped with appropriate weights of Cu(NO_3_)_2_ 3H_2_O and Zn(NO_3_)_2_ 6H_2_O. A diluted aqueous ammonia solution was added dropwise to prepare the precipitate, and the mixture was constantly stirred for half an hour, or until pH 8. The resultant precipitate was dried, washed several times with deionized water, and then calcined at 600 °C for 3 h.^[^ ^30^ ^]^

#### 2.4.2 Pure, Mono, and Dual TiO_2_ Nanoparticle Characterization

The X‐ray diffraction (XRD) method (PANalytical X‐ray diffraction apparatus type X′Pert PRO) was used to characterize the NPs. The XRD technique is based on the constructive interference of monochromatic X‐rays with a crystalline material. X‐rays, or electromagnetic radiation with shorter wavelengths, are produced when electrically charged particles with sufficient energy slow down. Pure TiO_2_. Ti0.985Cu0.015O2 Ti0.97Cu0.015Zn0.015O2 particles are the target of the collimated X‐rays generated in XRD. The interaction of the incident rays with the sample results in a diffracted ray, which is then detected, processed, and counted. Plotting the intensity of the diffracted rays dispersed at different angles of the material shows a diffraction pattern.

The samples were characterized using a diffuse reflectance method (JASCO, model V‐570 UV–vis–NIR) and a transmission electron microscope (TEM, JEOL JEM‐2100). After being diluted with distilled water, the resulting pure Ti0.985Cu0.015O2 Ti0.97Cu0.015Zn0.015O2 was sonicated for three minutes in an ultrasonic bath. Five microliters of each sample was then put on a 200‐mesh grid covered with film and left for ten minutes. Finally, TEM was applied to the grids.

### 2.5 Adipose Tissue Stem Cells CM Preparation

Trypsinization was performed on the second passage of AD‐MSCs at around 80% confluence cells using 0.25% trypsin and 0.01% ethylenediaminetetraacetic acid (EDTA) in PBS for 5 min at 37 °C. The cells were centrifuged, then resuspended in DMEM medium with 10% FBS and 1% penicillin‐streptomycin, and then incubated in a 75 cm^2^ culture flask.^[^ ^56^ ^]^ After the cells were starved on day three, the old complete media was taken out and replaced with serum‐free, low‐glucose DMEM media that included 1% penicillin‐streptomycin. This allowed the cells’ secretome and cytokines to be released. We gathered material into a 15 mL tube on day five. In a centrifuge set at 4 ºC, centrifugation was carried out for 10 min at 2,000 rpm. CM, the supernatant, was preserved and utilized in subsequent studies^[^ ^31^ ^]^

### 2.6 Groups

Control group (CT): AD‐MSCs treated with CM. LPS group: AD‐MSCs treated with LPS dissolved in complete media for induction of inflammation. TiO_2_ nanoparticle group (Ti): AD‐MSCs treated with TiO_2_ nanoparticles suspended in CM. Mono‐doped TiO_2_ nanoparticle group (Ti mono): AD‐MSCs treated with TiO_2_Cu nanoparticles suspended in conditional media (CM). Dual‐doped TiO_2_ nanoparticle group (Ti dual): AD‐MSCs treated with TiO_2_ Cu Zn nanoparticles suspended in CM.

### 2.7 Cytotoxicity Using MTT Assay

To test the safety of the TiO_2_‐based nanoparticles, the cytotoxicity of control (CM), LPS, TiO_2_ nanoparticle + LPS, mono‐doped TiO_2_ nanoparticle + LPS, and dual‐doped TiO_2_ nanoparticle + LPS on stem cells isolated from adipose tissue (AD‐MSCs) by MTT assay was performed. The AD‐MSCs were extracted by trypsinization, cleaned in PBS, and plated with 96‐well plates before testing. The cells were cultivated at 37 °C with 5% CO_2_ for 24 h. The cells were treated to 0, 20, 40, 60, 80, and 100 μg mL^−1^ of the recommended nanoparticles and LPS after a 24‐hour incubation period. Cell growth is measured by the capacity of live cells to convert the yellow dye 3‐(4,5‐dimethyl‐2‐thiazolyl)‐2,5‐diphenyl‐2H‐terazolium bromide (MTT) into a blue formazan product.^[^ ^53^ ^]^ MTT solution was added to the medium in each well after a 24‐hour incubation period, and the plates were then incubated for four hours at 37 ºC with 5% CO_2_. The formazan crystals from the living cells were dissolved in DMSO and agitated vigorously after the MTT reagent was removed. An ELISA reader was then used to measure the absorbance at 492 nm.^[^ ^32^ ^]^

### 2.8 Oxidative Markers

The level of peroxidation of lipid was evaluated via MDA in addition to nitric oxide (NO) measurements using spectrophotometry kits purchased from Biodiagnostic and were performed according to manufacturer instructions. These markers were measured using a colorimetric method at 532 nm.

### 2.9 TAC Assay

Following the manufacturer's instructions, the ferric reduction antioxidant power technique was used to measure the quantity of TAC in the samples using a spectrophotometry kit that was obtained from Biodiagnostic. The Fe‐3‐TPTZ complex is reduced to generate a vivid blue hue at 593 nm.

### 2.10 ELISA

ELISA kits purchased from Elabscience (China) were used to assay the test samples for interleukins IL4 and IL6, TNF‐α, and lipoxygenase inhibition activity (5‐LOP) in accordance with manufacturer instructions. The absorbance of the reaction solutions was measured using a microplate reader.

### 2.11 Protein Denaturation

A protein denaturation test was conducted using the Gambhire et al. technique,^[^ ^33^ ^]^ with some modifications as described in Gunathilake et al.^[^ ^34^ ^]^ The reaction mixture (5 mL) was made up of 0.02 mL of the tested sample, 4.78 mL of PBS (pH 6.4), and 0.2 mL of 1% bovine albumin. After combining the mixture, it was incubated for 15 min at 37 °C in a water bath before being heated for 5 min at 70 °C. After cooling, the turbidity was measured at 660 nm using a UV spectrometer. The percentage of protein denaturation inhibition was calculated.

### 2.12 Proteinase Inhibitory Activity

The test sample's proteinase inhibitory activity was assessed using Sakat et al.'s methodology,^[^ ^35^ ^]^ which is modified by Gunathilake et al.^[^ ^34^ ^]^ To be specific, the reaction mixture (2 mL) included 1 mL of test material, 0.06 mg of trypsin, and 1 mL of 20 mM Tris‐HCl buffer (pH 7.4). One milliliter of 0.8% (w/v) casein was added after the mixture had been incubated for 5 min at 37 °C. It was then incubated for a further 20 min. After the incubation time, the process was stopped by applying 2 mL of 70% perchloric acid. After the mixture was centrifuged, the absorbance of the supernatant was measured at 210 nm with buffer serving as the reference. The percentage of protein denaturation inhibition was calculated.

### 2.13 Determination of Antioxidant Activities

The hydroxyl radical scavenging activity was carried out as follows: 2.4 mL of phosphate buffer (pH 7.8) was added to test tubes to make the reaction mixture. For the test sample and the standard (L‐ascorbic acid) at different concentrations (100%, 10%, 1%, 0.1%, and 0.01%), 90 μL of 1 mM 1, 10 phenanthroline, 150 μL of 0.1 mM hydrogen peroxide, 60 μL of 1 mM iron (III) chloride, and 5 min of room temperature incubation were needed, excluding the controls. Based on the observed increase in absorbance at 560 nm, a radical scavenging activity estimate was performed.

1,1 diphenyl‐2‐picrylhydrazyl (DPPH) was used in the DPPH radical scavenging experiment in accordance with the procedure outlined by Brand‐Williams et al.^[^ ^36^ ^]^ with some modifications. In conclusion, the following concentrations of L‐ascorbic acid were generated: 0.0625, 0.125, 0.25, 0.5, and 1 mg mL^−1^. As a rule, this antioxidant was used. One milliliter was measured for each test sample. Then 0.5 mL of 0.3 mM DPPH in methanol was added to a clean test tube. The mixture was shaken thoroughly and then let to stand at room temperature for 15 min in the absence of light. Blank solutions comprising the test sample (2.5 mL) and 1 mL of methanol were used as a reference. The negative control consisted of 1 mL of methanol and 2.5 mL of DPPH solution, while the positive control was L‐ascorbic acid at the same concentration as the test sample. Following a dark incubation period, the absorbance values were recorded at 517 nm using a spectrophotometer. The DPPH radical scavenging activity was estimated.

### 2.14 Genetic Expressions

qRT‐PCR was used to measure the gene expression levels of glutathione peroxidase (GPx), catalase (CAT), superoxide dismutase 1 (SOD1), interleukin 4 (IL‐4), interleukin 6 (IL‐6), and TNF‐α in AD‐MSCs, along with beta‐actin (β‐Actin) as a reference gene. As directed by the manufacturer, total RNA was extracted using the RNeasy Mini kit. The amount and kind of RNA produced were identified. Equal amounts of extracted RNA were then reverse‐transcribed into complementary DNA (cDNA) using a cDNA synthesis kit in accordance with the manufacturer's instructions. The genes’ mRNA expression was assessed using qRT‐PCR using the manufacturer's suggested SYBR green PCR Master Mix. In **Table** **1**, the primer sequence is explained. In the thermal cycler technique, 45 cycles of 5 s denaturation at 95 °C and 10 s annealing/extension at 60 °C were performed after the first 5 min of enzyme activation at 95 °C. The target gene expression changes were calculated using the ΔΔCT technique and reported as fold change (FC = 2 − ΔΔCT). The housekeeping gene, β‐actin, was used to normalize each measurement.

**Table 1:** qRT‐PCR primers sequence (5′ to 3′) that was used for gene expression analysis.

Gene	Sequence [5′ to 3′]
GPx	Forward: CACAGTCCACCGTGTATGCC Reverse: AAGTTGGGCTCGAACCCACC
CAT	Forward: GTCCGATTCTCCACAGTCGC Reverse: CGCTGAACAAGAAAGTAACCTG
SOD1	Forward: ATGTGTCCATTGAAGATCGTGTGA Reverse: GCTTCCAGCATTTCCAGTCTTTGTA
IL‐4	Forward: TGCACCGAGATGTTTCC Reverse: GGATGCTTTTTAGGCTTTCC
IL‐6	Forward: GCCCTTCAGGAACAGCTATGA Reverse: TGTCAACAACATCAGTCCCAAGA
Beta‐actin	Forward: CCCATCTATGAGGGTTACGC Reverse: TTTAATGTCACGCACGATTTC
TNF‐α	Forward: AAATGGGCTCCCTCTCATCAGTTC Reverse: TCTGCTTGGTGGTTTGCTACGAC

### 2.15 Statistical Analysis

Each experiment was conducted 3 independent times (n = 3). The mean ± standard deviation (SD) from three independent experiments was conducted in this study. Statistical analyses were carried out using SPSS 18.0 software. Comparisons between groups were evaluated using one‐way ANOVA, with significance considered; at a: significant difference versus CT at (*p* < 0.05). b: significant difference versus LPS at (*p* < 0.05). c: significant difference versus LPS at (*p* < 0.01).

## 3 Results

### 3.1 Characterization of AD‐MSCs

**Figure 1:**
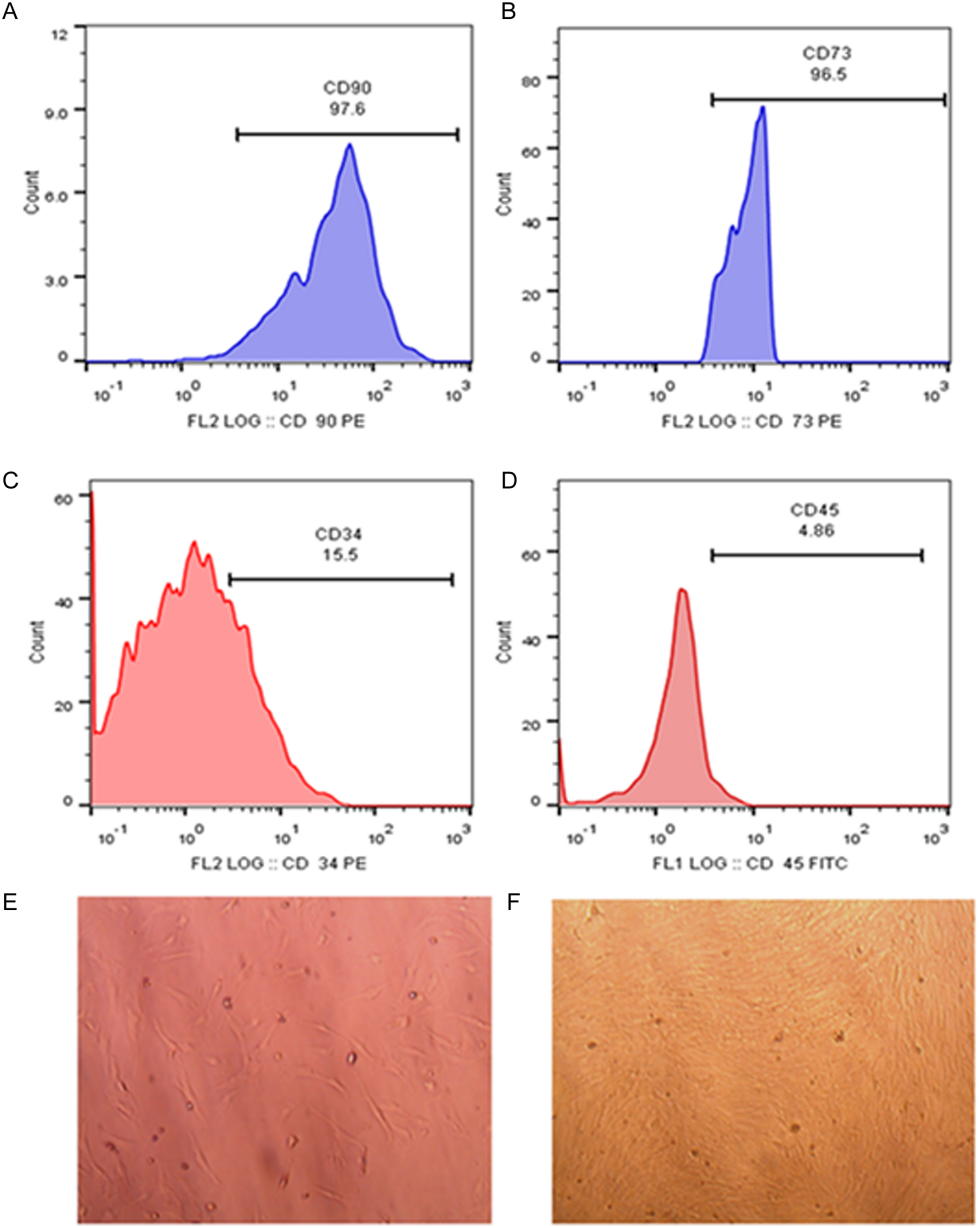
**AD‐MSCs characterization.** A–D) CD90, CD73, CD34, and CD45 identification of AD‐MSCs using flow cytometry. E) AD‐MSCs 3 days after isolation. F) AD‐MSCs complete sheet.

**Figure** **1** shows AD‐MSCs characterization. Positive CD markers (CD90 and CD73) were overexpressed on the cell surfaces of AD‐MSCs recording 97.6% and 96.5%, respectively, using flow cytometry as shown in Figure 1(A,B). On the other side, negative CD markers (CD34 and CD45) were 15.5% and 4.86%, respectively, on the cell surfaces of AD‐MSCs using flow cytometry as shown in Figure 1(C,D). Figure 1 (E,F) shows the bright field microscopy of the isolated AD‐MSCs after 3 days then once the complete sheet is achieved.

### 3.2 Nanoparticles Characterization

**Scheme 1:**
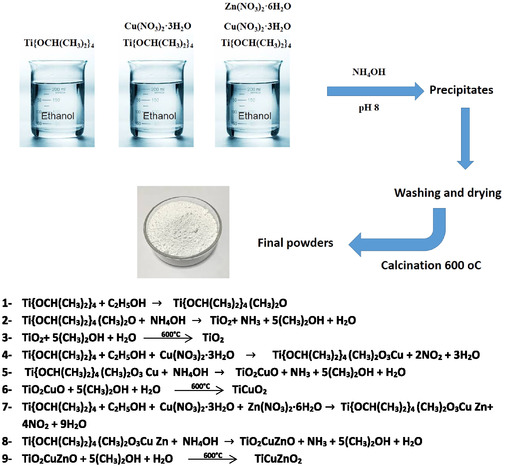
Diagram for the formation of nanoparticles and the equations for the reactions that took place to obtain the nanoparticles. The obtained nanoparticles color change was TiO_2_ white color, Ti0.97Cu0.03O2, and Ti0.97Cu0.015Zn0.015O2 beige color. The preparation steps we mentioned in the equation such as temperature, continuous steering, and precipitation. In addition to the accuracy in the preparation based on the protocols for nanomaterial preparations also we followed the personal protective equipment (PPE) including protective clothing, helmets, goggles, or other garments or equipment designed to protect the wearer's body from hazards.

Diagram for the formation of nanoparticles and the equations for the reactions that took place to obtain the nanoparticles illustrated in **Scheme** **1**.

**Figure 2:**
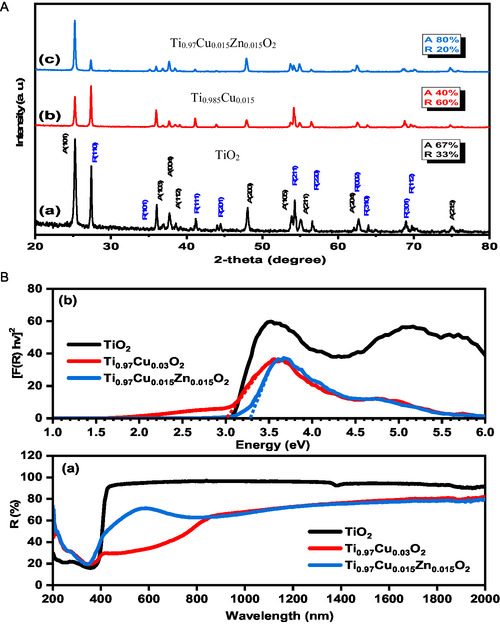
**XRD and bandgap of the NPs.** A) XRD patterns of a) pure TiO_2_, b) Ti_0.985_Cu_0.015_O_2_, and c) Ti_0.97_Cu_0.015_Zn_0.015_O_2_ powders. B) a) diffuse reflectance and b) optical bandgap of pure TiO_2_, Ti_0.97_Cu_0.03_O_2_, and Ti_0.97_Cu_0.015_Zn_0.015_O_2_ samples.

**Figure** **2**A demonstrates the XRD patterns of pure TiO_2_, Ti_0.985_Cu_0.015_O_2_, and Ti_0.97_Cu_0.015_Zn_0.015_O_2_ powders synthesized by the coprecipitation technique. The XRD shape of the obtained pure TiO_2_ powder displayed the fabrication of mixed phases indexed to anatase TiO_2_ (67%, JCPDS, 84‐1286) and rutile (33%, JCPDS 21‐1276). The incorporation of Cu and (Cu, Zn) ions into the TiO_2_ structure leads to sensible variations in the ratio of anatase to rutile phase. The mono doping by Cu^2+^ ions induces a change in anatase to rutile ratio to 40% and 60%, respectively. For (Cu, Zn) codoping, the ratio of anatase structure was significantly improved to 80% whereas the ratio of rutile structure was decreased to 20%. As a result, through regulating the doping process, a preferred ratio of anatase or rutile TiO_2_ can be produced. In the XRD patterns, there are no extra XRD peaks linked to any impurities were detected. The size of the pure TiO_2_, Ti_0.985_Cu_0.015_O_2_, and Ti_0.97_Cu_0.015_Zn_0.015_O_2_, powders was calculated built on Scherrer's Equation (1):

(1) D=0.9λ/βcos θ

The undoped TiO_2_ sample has an average crystallite size = 56 nm. The crystallite size was increased to 63 and 66 nm after incorporation of Cu and (Cu, Zn) ions, respectively.

Figure 2B demonstrates the variation in the strength of the diffuse reflectance with wavelength (nm) for pure TiO_2_, Ti_0.97_Cu_0.03_O_2_, and Ti_0.97_Cu_0.015_Zn_0.015_O_2_ samples (200–2000 nm). It can be seen that the Ti_0.97_Cu_0.03_O_2_ and Ti_0.97_Cu_0.015_Zn_0.015_O_2_ samples have low‐intensity values related to the pure one. As well, the sharp absorption edge of pure TiO_2_ was extended over more wavelength ranges after Cu doping. These points recommended that the Ti_0.97_Cu_0.03_O_2_ sample has high absorption properties compared to the pure sample. The Kubelka–Munk (K–M) equation, F(R) = (1‐R)^2^/2R = K/S, was used to assess the precise value of the bandgap energy of the manufactured TiO_2_, Ti_0.97_Cu_0.03_O_2_ and Ti_0.97_Cu_0.015_Zn_0.015_O_2_ samples, as shown in Figure 2(b). the bandgap energy of TiO_2_, Ti_0.97_Cu_0.03_O_2_, and Ti_0.97_Cu_0.015_Zn_0.015_O_2_ samples was measured to be 3.1, 3, and 3.3 eV, correspondingly. The red changes in the bandgap of the Ti_0.97_Cu_0.03_O_2_ lattice are commonly linked to the s‐d and p‐d interaction of the parent and dopant ions. The introduction of Zn ions plus Cu ions sensibly blue shifts the band gap energy of the TiO_2_ sample.

**Figure 3:**
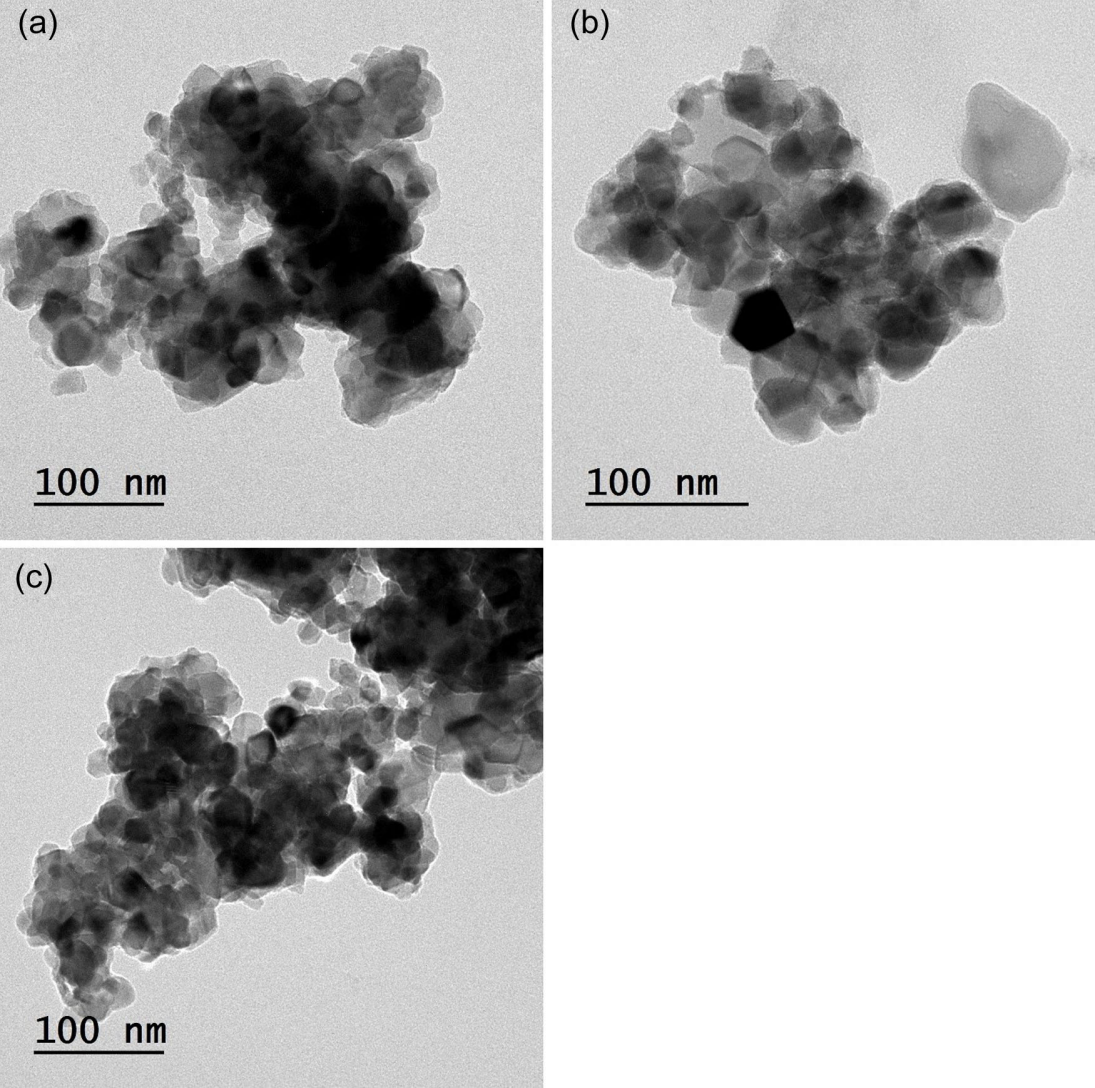
TEM images of a) TiO_2_, b) Ti_0.985_Cu_0.015_O_2_, and c) Ti_0.97_Cu_0.015_Zn_0.015_O_2_ powders.

The TEM pictures of TiO_2_, Ti_0.985_Cu_0.015_O_2_, and Ti_0.97_Cu_0.015_Zn_0.015_O_2_ are illustrated in **Figure** **3**a–c. TEM images of the powders display the construction of nanoparticles that have attached or overlapped together. The greatest of the nanoparticles possessing asymmetrical shape with minor nanoparticles possess approximately sphere‐shaped. The mean particles size of TiO_2_, Ti_0.985_Cu_0.015_O_2_, and Ti_0.97_Cu_0.015_Zn_0.015_O_2_ nanoparticles was measured to be 29, 35, and 33 nm, respectively.

**Figure 4:**
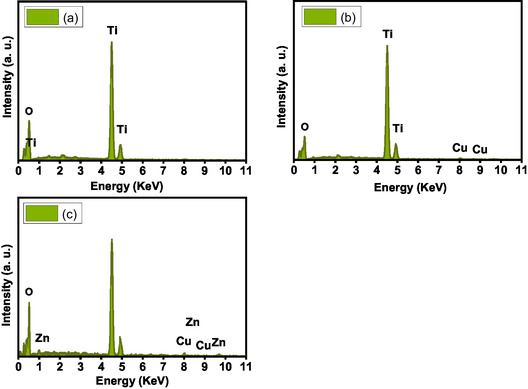
EDX pattern of a) pure, b) Cu doped ), and c) (Zn, Cu) codoped TiO_2_ samples.

**Figure** **4** depicts the energy‐dispersive X‐ray analysis (EDX) pattern of pure, Cu doped, (Zn, Cu) codoped TiO_2_ samples synthesized by the coprecipitation method. The EDX patterns confirm the presence of Ti, O, Zn, and Cu elements without the appearance of any other elements inside the patterns, suggesting high purity of these compositions with desired chemical structures. With detection error of EDX analysis, the computed weights of Zn and Cu elements are near those used in the preparation step which supports the synthesis of anticipated compositions. The detected quantity of Cu dopant in Cu doped is 1.16 wt%. In case of (Zn, Cu) codoped TiO_2_ sample, detected quantities of Cu and Zn dopants are 1.4 and 0.95 wt%, respectively.

### 3.3 Cytotoxicity

**Figure 5:**
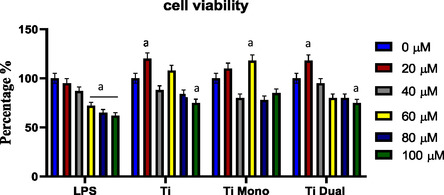
Cell viability of AD‐MSCs following induction with LPS, treatment with TiO_2_ alone (Ti), TiO_2_ mono doped with copper (Ti Mono), TiO_2_ dual doped with copper and zinc (Ti Dual). a: significant difference versus CT at (*p* < 0.05).

Cell viability of AD‐MSCs was observed following induction with LPS and treatment with increasing doses of TiO2, mono TiO_2_, or dual TiO_2_ (0, 20, 40, 60, 80, and 100 μg mL^−1^). **Figure** **5** shows that there was a significant cytotoxic effect (*p* > 0.05) against AD‐MSCs reaching around 40% cell death at 100 μg mL^−1^ LPS treatment. In contrast, there were nonsignificant variations (*p* < 0.05) in the cytotoxic effects of the LPS‐treated cells with TiO_2_, mono TiO_2_, or dual TiO_2_.

### 3.4 Oxidative Modulation

**Figure 6:**
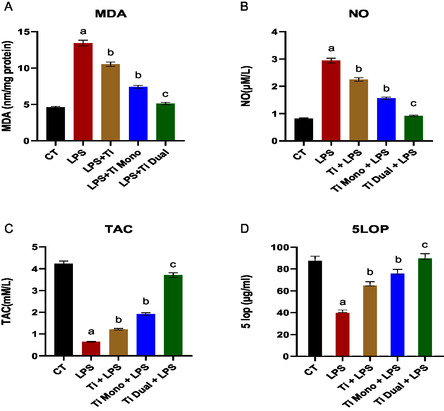
Oxidative markers. A) MDA, B) NO, C) TAC, and D) 5‐LOP levels in CM of AD‐MSCs treated with LPS, TiO_2_ to which LPS was added, mono‐doped TiO_2_ to which LPS was added (Ti mono + LPS), and dual‐doped TiO_2_ to which LPS was added (Ti dual + LPS). a: significant difference versus CT at (*p* < 0.05). b: significant difference versus LPS at (*p* < 0.05). c: significant difference versus LPS at (*p* < 0.01).

ROS production is responsible for AD‐MSCs differentiation and plays a serious part in the initiation of key inflammatory signaling pathways. In this study, we measured the capability of different TiO_2_‐based nanoparticles in AD‐MSCs stimulated by LPS. MDA and NO levels are two important ROS markers. Induction of the AD‐MSCs with LPS high significantly increased the MDA and NO levels paralleled to untreated control (*p* > 0.01). The LPS‐treated AD‐MSCs with TiO_2_ and mono TiO_2_ nanoparticles significantly reduced the MDA and NO levels (*p* > 0.05), while dual TiO_2_ nanoparticles high significantly reduced the MDA and NO levels (*p* > 0.01) compared to induced cells with LPS (**Figure** **6**A,B).

TAC and 5‐LOP activity were monitored to investigate the scavenging capacity of the proposed nanoparticles toward ROS. Induction of the AD‐MSCs with LPS high significantly decreased the TAC and 5‐LOP activity compared to untreated control (*p* > 0.01). The LPS‐treated AD‐MSCs with TiO_2_ and mono TiO_2_ nanoparticles significantly increased the TAC and 5‐LOP activity (*p* > 0.05), while dual TiO_2_ nanoparticles high significantly increased the TAC and 5‐LOP activity (*p* > 0.01) compared to induced cells with LPS (Figure 6C,D).

**Figure 7:**
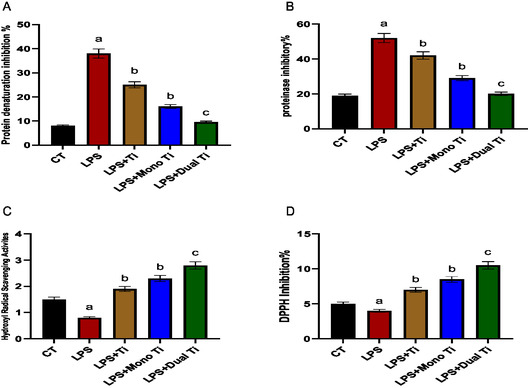
Oxidative modulators. A) Protein denaturation inhibition percentage, B) proteinase inhibitory percentage, C) hydroxyl radical scavenging activity, and D) DPPH inhibition percentage in CM of AD‐MSCs treated with LPS, TiO_2_ to which lipopolysaccharide was added, mono‐doped TiO_2_ to which LPS was added (Ti mono + LPS), and dual‐doped TiO_2_ to which LPS was added (Ti dual + LPS). a: significant difference versus CT at (*p* < 0.05). b: significant difference versus LPS at (*p* < 0.05). c: significant difference versus LPS at (*p* < 0.01).

Protein denaturation inhibition and proteinase inhibitory percentages were investigated in the AD‐MSCs upon different treatments. Induction of the AD‐MSCs with LPS high significantly increased the protein denaturation inhibition and proteinase inhibitory percentages compared to untreated control (*p* > 0.01). The LPS‐treated AD‐MSCs with TiO_2_ and mono TiO_2_ nanoparticles significantly decreased the protein denaturation inhibition and proteinase inhibitory percentages (*p* > 0.05), while dual TiO_2_ nanoparticles high significantly decreased the Protein denaturation inhibition and proteinase inhibitory percentages (*p* > 0.01) compared to induced cells with LPS (**Figure** **7**A,B).

Hydroxyl radical scavenging activity and DPPH inhibition percentage were monitored in AD‐MSCs upon the proposed treatments. Induction of the AD‐MSCs with LPS high significantly decreased the hydroxyl radical scavenging activity and DPPH inhibition percentage compared to untreated control (*p* > 0.01). The LPS‐treated AD‐MSCs with TiO_2_ and mono TiO_2_ nanoparticles significantly increased the hydroxyl radical scavenging activity and DPPH inhibition percentage (*p* > 0.05), while dual TiO_2_ nanoparticles high significantly increased the hydroxyl radical scavenging activity and DPPH inhibition percentage (*p* > 0.01) compared to induced cells with LPS (Figure 7C,D).

### 3.5 Interleukins and TNF‐α

**Figure 8:**
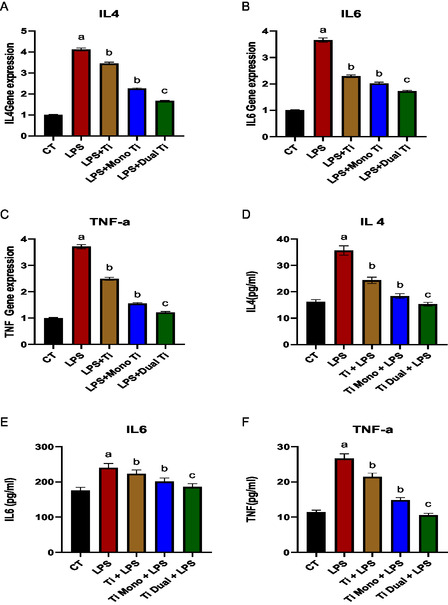
Genetic and protein expression levels of interleukins and TNF‐α. A,B) Genetic expression levels of interleukins‐4, 6 and C) TNF‐α. D,E) Protein expression levels of interleukins‐4, 6 and F) TNF‐α in CM of AD‐MSCs treated with LPS, TiO_2_ to which lipopolysaccharide was added, mono‐doped TiO_2_ to which LPS was added (Ti mono + LPS), and dual‐doped TiO_2_ to which LPS was added (Ti dual + LPS). a: significant difference versus CT at (*p* < 0.05). b: significant difference versus LPS at (*p* < 0.05). c: significant difference versus LPS at (*p* < 0.01).

Many studies have inveterate that interleukins are crucial pro‐inflammatory cytokines and TNF‐α is significant pro‐inflammatory mediator in inflammation processes. Genetic and protein expression levels of interleukins and TNF‐α were assessed in AD‐MSCs upon treatments with TiO_2_, mono TiO_2_, and dual TiO_2_ nanoparticles. Induction of the AD‐MSCs with LPS high significantly unregulated the interleukins‐4, 6 and TNF‐α in the genetic and protein levels paralleled to the untreated control (*p* > 0.01). The LPS‐treated AD‐MSCs with TiO_2_ and mono TiO_2_ nanoparticles significantly decreased the interleukins‐4, 6, 10, and TNF‐α in the genetic and protein levels (*p* > 0.05), while dual TiO_2_ nanoparticles high significantly decreased the interleukins‐4, 6, and TNF‐α in the genetic and protein levels (*p* > 0.01) compared to induced cells with LPS (**Figure** **8**A–F).

### 3.6 Antioxidant Capacity

**Figure 9:**
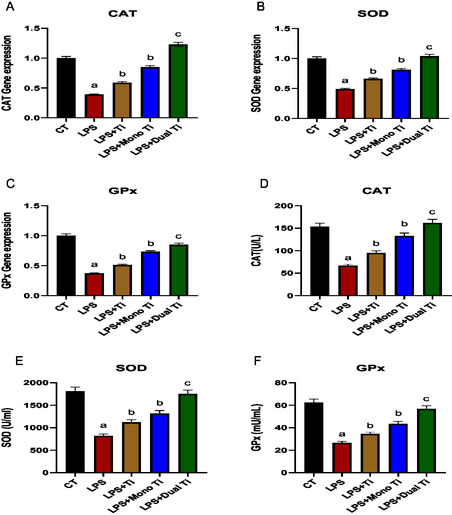
Genetic and protein expression levels of CAT, SOD, and GPx. A–C) Genetic expression levels of CAT, SOD, and GPx. D–F) Protein expression levels of CAT, SOD, and GPx in CM of AD‐MSCs treated with LPS, TiO_2_ to which lipopolysaccharide was added, mono‐doped TiO_2_ to which LPS was added (Ti mono + LPS), and dual‐doped TiO_2_ to which LPS was added (Ti dual + LPS). a: significant difference versus CT at (*p* < 0.05). b: significant difference versus LPS at (*p* < 0.05). c: significant difference versus LPS at (*p* < 0.01).

Genetic and protein expression levels of CAT, SOD, and GPx were assessed in AD‐MSCs upon treatments with TiO_2_, mono TiO_2_, and dual TiO_2_ nanoparticles. Induction of the AD‐MSCs with LPS high significantly downregulated the CAT, SOD, and GPx in the genetic and protein levels compared to the untreated control (*p* > 0.01). The LPS‐treated AD‐MSCs with TiO_2_ and mono TiO_2_ nanoparticles significantly upregulated the CAT, SOD, and GPx in the genetic and protein levels (*p* > 0.05), while dual TiO_2_ nanoparticles high significantly upregulated the CAT, SOD, and GPx in the genetic and protein levels (*p* > 0.01) compared to induced cells with LPS (**Figure** **9**A–F).

## 4 Discussion

Our work provides evidence that the conditional media of the adipose stem cells (MSC‐CM) when supplemented with titanium dioxide nanoparticles (Ti, Ti mono, Ti dual) play a major part in meaningfully reducing LPS‐mediated inflammation through suppression of reactive oxygen species (MDA and NO) levels, downregulation of interleukins (IL4 and IL6) and TNF‐α expression levels as well as increase the TAC, 5‐LOP, CAT, SOD1, GPx activities, and DPPH inhibition percentage. Further, the current observations were in agreement with prior work indicating that MSCs suppress inflammation and oxidation through modulation of JNK and ERK1/2, increase the appearance of heme oxygenase‐1, and downregulation of the NF‐kB.^[^ ^37^, ^38^ ^]^

The MSC‐CM reduced the synthesis of pro‐inflammatory cytokines such as IL1β and IL6 in cells activated by LPS. This demonstrated that MSC‐CM treatment reduced the inflammatory components produced by LPS‐stimulated cells by blocking the mRNA transcription of pro‐inflammatory cytokines and chemokines.^[^ ^39^, ^40^ ^]^

The last‐mentioned observations indicated the importance of MSC‐CM as an anti‐inflammatory candidate. MSC‐CM contains the entire secretome of stem cells, which is defined as the growth factors, regulatory proteins, cytokines, and extracellular vesicles secreted into their extracellular matrix.^[^ ^41^ ^]^ The immunomodulatory chemicals found in the secretome play a major role in mediating the effects of MSC‐CM, and the final effects are mostly determined by the ratio of pro‐inflammatory to anti‐inflammatory cytokines.^[^ ^55^ ^]^ IL10, TNF‐b, and MSC‐CM are anti‐inflammatory chemicals that have been demonstrated to inhibit inflammatory genes, including TNF‐α.^[^ ^42^ ^]^ As such, several applications involving the anti‐inflammatory role of MSC‐CM have emerged, including their ability to suppress inflammatory genes associated with skin injuries caused by radiation.^[^ ^31^ ^]^ As well as their ability to attenuate inflammation involved in tendinopathy.^[^ ^43^ ^]^ MSC‐CM also possesses antioxidant capacity, as evidenced by their ability to suppress reactive oxygen species generation in the retinal damage model.^[^ ^44^ ^]^ MSC‐CM are also highly resistant to lipid peroxidation and the production of lipid ROS.^[^ ^45^ ^]^

Gram‐negative bacteria's outer membrane contains LPS, which is referred to as an endotoxin since it can induce systemic inflammation. LPS induce inflammation by stimulating toll‐like receptor which recognizes a variety of pathogens and invading substances. Upon stimulation, toll‐like receptor activates signaling pathways of various in nanoparticles significantly inflammatory cytokines, type 1 interferon, and numerous inflammatory mediators. This process is essential for normal immune response, but overactivation by LPS leads to systemic inflammation.^[^ ^46^ ^]^ Because the activation of the toll‐like receptor increases the activity of NADPH oxidase isoform 2 (NOX2), which produces ROS and depletes antioxidant enzymes, primarily CAT, SOD, and GPx, LPS can also cause oxidative.^[^ ^47^ ^]^

The study found that TiO_2_ dual doped with Cu and Zn suspended in MSC‐CM had the highest antioxidant capacity, followed by TiO_2_ mono doped with Cu suspended in MSC‐CM and, finally, TiO_2_ alone suspended in MSC‐CM. All of them significantly decreased the oxidative marker MDA while boosting the activity of the antioxidant enzymes CAT, GPx, and SOD. They could accomplish this by triggering the nuclear factor erythroid 2‐related factor 2 (Nrf2) pathway, which lowers oxidative by stimulating heme oxygenase 1 (HO‐1).^[^ ^48^, ^49^ ^]^

Under typical circumstances, Kelch‐like ECH‐associated protein 1 (keap1) uses sulfide bonds to bind (Nrf2) in the cytoplasm. Keap1 is in charge of the quick breakdown and sequestration of (Nrf2) in physiological conditions. But upon oxidative mediated by various insults such as radiation or toxemia, (Nrf2) is liberated from keap1 and subsequent phosphorylation allows for increased half‐life and migration into the nucleus. (Nrf2) upregulates several cytoprotective genes, including those responsible to produce GPx, glutathione reductase which reduces oxidized glutathione into the sulfhydryl (SH) form, SOD, CAT. Among the proteins regulated by (Nrf2) is HO‐1, which is responsible for the generation of iron and subsequently ferritin, biliverdin, and then bilirubin which are both antioxidants in nature. Thus the Nrf2/HO‐1 pathway activation plays an important role in the antioxidant action.^[^ ^50^ ^]^

A family of transcription factors known as Nf‐kb is involved in both physiological and pathological processes, among other circumstances. The canonical and noncanonical routes are the two Nf‐kb activation pathways that have been identified. While the noncanonical route is involved in relatively specialized functions, the canonical pathway's significance in immunity and inflammatory response has been established.^[^ ^54^ ^]^ In short, Nf‐kb is ordinarily attached to IkB kinases (IKK). However, when these inhibitory kinases are activated by external inflammatory stimuli, they get phosphorylated, which is then followed by proteasome destruction. Consequently, the Nf‐kb is freed and may go into the nucleus. Next, Nf‐kb triggers several genes that produce inflammatory cytokines and compounds, including TNF‐a and IL6.^[^ ^51^ ^]^

Anti‐inflammatory activity was also observed; being highest with dual‐doped TiO_2_ followed by mono‐doped TiO_2_ and lastly TiO_2_ alone. These compounds were able to reverse LPS‐induced increase in IL4, IL6, and IL10 through action on NF‐Kb, while they were able to lower the concentration of NO through reduced expression of iNOS.^[^ ^52^ ^]^ To confirm these results and their underlying mechanisms, molecular assays were carried out to measure the expression level of genes responsible for the production of inflammatory and oxidative markers. Genes used to test oxidative were SOD, GPx, and CAT, while genes used to test inflammation were IL4, IL6, and TNF‐α.

According to the results, these genes’ expression increased following LPS induction as compared to control, suggesting oxidative and inflammation. When compared to LPS, all treatments significantly decreased gene expression; dual‐doped TiO_2_ had the largest drop, followed by mono‐doped TiO_2_ and finally TiO_2_ alone. These findings support the anti‐inflammatory and antioxidant properties of TiO_2_, mono TiO_2_, and dual TiO_2_ at the mechanistic level and are consistent with the findings of measures of oxidative and inflammation biomarkers. In addition, the compatibility of TiO_2_, mono TiO_2_, and dual TiO_2_ was evaluated by cytotoxicity tests. Cell viability of adipose tissue‐derived stem cells after incubation with LPS, TiO_2_, mono TiO_2_, and dual TiO_2_ was determined. All treatments achieved significant elevation of cell viability compared to LPS, indicating compatibility of the mentioned treatment on a cellular level.

## 5 Conclusion

It was concluded that dual‐doped TiO_2_ nanoparticle suspended in stem cells conditional media was the best candidate for anti‐inflammatory/antioxidant ability that was obvious in the measurement of inflammatory markers and in oxidative markers as well as in biochemical assays that were confirmed by genetic expressions. Through the suppression of reactive oxygen species (MDA and NO), downregulation of interleukins (IL4 and IL6), and downregulation of TNF‐α expression levels, this therapeutic approach may be crucial in reducing LPS‐mediated inflammation. It may also increase the TAC, 5‐LOP, CAT, SOD1, GPx activities, and the percentage of DPPH inhibition. The dual‐doped TiO_2_ nanoparticle's capacity to lower inflammation and oxidative may find additional use in an in vivo experimental paradigm.

## 6 Limitations of Study

The study's limitations include its in vitro design, which may not fully replicate in vivo conditions, and the use of AD‐MSCs derived from Sprague Dawley rats, which could limit the generalizability to human cells or clinical applications. The focus on specific TiO_2_, mono‐doped, and dual‐doped TiO_2_ nanoparticles may overlook the effects of other nanomaterials with different properties. Cytotoxicity was assessed using the MTT assay, which might not capture all toxic effects or long‐term impacts of the nanoparticles. Additionally, while several oxidative and inflammatory markers were analyzed, the study may not account for other relevant pathways or markers associated with inflammation and oxidative stress.

## Conflict of Interest

The authors declare no conflict of interest.

## Author Contributions

All authors contributed to this article are as follows **Ahmed A. Abd‐Rabou**: and **Mohamed S. Kishta**: hypothesized the article main idea, biochemical and genetic analyses, wrote the main manuscript text, and prepared the figures. **Ahmed M. Youssef**: prepared and characterized the nanoparticles. **Mohamed I. El‐Khonezy**: and **Soheir E. Kotob**: measured the anti‐inflammatory and antioxidant parameters.

## Data Availability

The data that support the findings of this study are available from the corresponding author upon reasonable request.
